# Supplementary material for: E7 Peptide Enables BMSC Adhesion and Promotes Chondrogenic Differentiation of BMSCs Via the LncRNA H19/miR675 Axis
Source: Bioengineering (Basel). 2023 Jun 30;10(7):781. doi: 10.3390/bioengineering10070781 (PMC10376115; doi:10.3390/bioengineering10070781)
Supplement: Supplementary file 1 [file bioengineering-10-00781-s001.zip › bioengineering-2416243-supplementary.pdf]

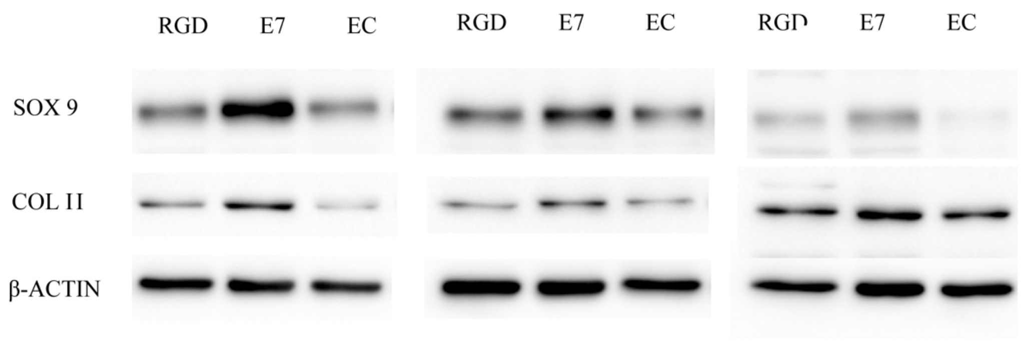

Original image of the subgraph 3B

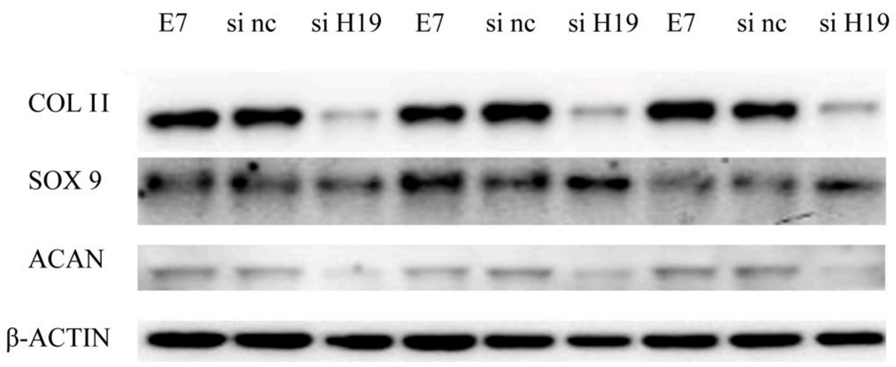

Original image of the subgraph 5C

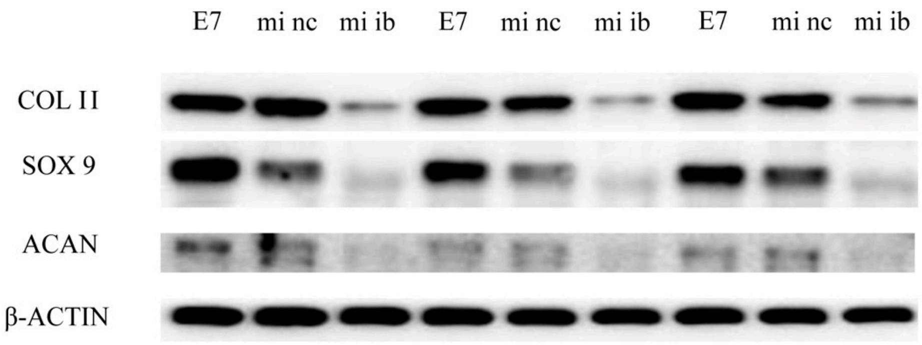

Original image of the subgraph 6B
